# Investigating the cause of a 2021 winter wave of COVID-19 in a border region in eastern Germany: a mixed-methods study, August to November 2021

**DOI:** 10.1017/S0950268824000761

**Published:** 2024-05-16

**Authors:** Buqing Yi, Eva Patrasová, Lenka Šimůnková, Fabian Rost, Sylke Winkler, Alexa Laubner, Susanne Reinhardt, Andreas Dahl, Alexander H. Dalpke

**Affiliations:** 1 Institute of Medical Microbiology and Virology, University Hospital Carl Gustav Carus, Technische Universität Dresden, Dresden, Germany; 2 Department of Epidemiology, Regional Public Health Authority for Ustecky Kraj, Ústí nad Labem, Czech Republic; 3 Third Faculty of Medicine, Charles University in Prague, Prague, Czech Republic; 4 DRESDEN-Concept Genome Center, Center for Molecular and Cellular Bioengineering, Technische Universität Dresden, Dresden, Germany; 5 Center for Regenerative Therapies Dresden, Technische Universität Dresden, Dresden, Germany; 6 Max Planck Institute of Molecular Cell Biology and Genetics, Dresden, Germany; 7 DRESDEN-Concept Genome Center, Technische Universität Dresden, Dresden, Germany; 8 Department of Infectious Diseases, Medical Microbiology and Hygiene, University of Heidelberg, Heidelberg, Germany

**Keywords:** community transmission, genomic epidemiology, genomic surveillance, pathogens, viral infection

## Abstract

It is so far unclear how the COVID-19 winter waves started and what should be done to prevent possible future waves. In this study, we deciphered the dynamic course of a winter wave in 2021 in Saxony, a state in Eastern Germany neighbouring the Czech Republic and Poland. The study was carried out through the integration of multiple virus genomic epidemiology approaches to track transmission chains, identify emerging variants and investigate dynamic changes in transmission clusters. For identified local variants of interest, functional evaluations were performed. Multiple long-lasting community transmission clusters have been identified acting as driving force for the winter wave 2021. Analysis of the dynamic courses of two representative clusters indicated a similar transmission pattern. However, the transmission cluster caused by a locally occurring new Delta variant AY.36.1 showed a distinct transmission pattern, and functional analyses revealed a replication advantage of it. This study indicated that long-lasting community transmission clusters starting since early autumn caused by imported or locally occurring variants all contributed to the development of the 2021 winter wave. The information we achieved might help future pandemic prevention.

## Introduction

To understand the impact of the COVID-19 pandemic and to design effective mitigation or prevention strategies, it is critical to decipher the transmissibility, prevalence and patterns of movement of SARS-CoV-2 infections, for which phylogenies have provided key information about the international spread of SARS-CoV-2 and enabled investigation of individual outbreaks and transmission chains [1, 2]. Phylodynamic approaches integrate evolutionary, demographic and epidemiological concepts and play an important role in tracking virus genetic changes, identifying emerging variants and informing public health strategy [3]. With these two powerful tools (phylogenetic analysis and phylodynamic analysis) genomic epidemiology may provide a lot of valuable information from several different aspects, from public health to important clinical parameters. To monitor the evolution of SARS-CoV-2 variants and to investigate transmission chains, since 2020 we have performed virus surveillance and genomic epidemiology research in Saxony, one state in Eastern Germany which neighbours the Czech Republic and Poland. To assist understanding about the context, here we present a brief introduction about the territorial division and some general characteristics of these places. Germany consists of 16 states with a total area of 357 588 km^2^ and a population of more than 83 million. Among the 16 states, Saxony is the tenth largest state by area (18 420 km^2^) and the sixth largest by population (around 4 million). Dresden is the capital city of Saxony, and other major cities include Leipzig and Chemnitz. Saxony is the only state in Germany which borders both the two central European countries of Poland and the Czech Republic with intense cross-border traffic. The Czech Republic has a total area of 78 871 km^2^, and a population of more than 10 million. The country has been divided into 14 administrative regions, among which ‘Ustecky Kraj’ shares the longest border with Saxony, Germany. The area of Ustecky Kraj is 5 339 km^2^ and this region has more than 0.8 million inhabitants, making it the fifth most populous region in the Czech Republic. ‘Usti nad Labem’ is the largest city in Ustecky Kraj. Poland has been divided into 16 states, with a total area of 312 696 km^2^, and a population of more than 38 million. Poland lies to the east of Saxony, and the Czech Republic lies to the south.

Through international collaboration, between 2021 March and 2022 March, with SARS-CoV-2 samples collected from a border region between Saxony, Poland and the Czech Republic, we performed genomic epidemiology analysis of these samples in a global background on a weekly basis. For identified virus mutant variants, active viruses were isolated and functional evaluations were performed to test their replication fitness and neutralization sensitivity against vaccine-elicited serum neutralizing antibodies. Thereby we previously identified a B.1.1.7 sub-lineage predominant in several European countries, such as Czech Republic, Austria, and Slovakia [4]. In addition to monitor virus evolution and transmission, with genomic epidemiology, we also analyzed why and how the pandemic developed in Saxony to evaluate the local pandemic or post-pandemic conditions, which may help predict what will take place in future.

**Figure 1.**
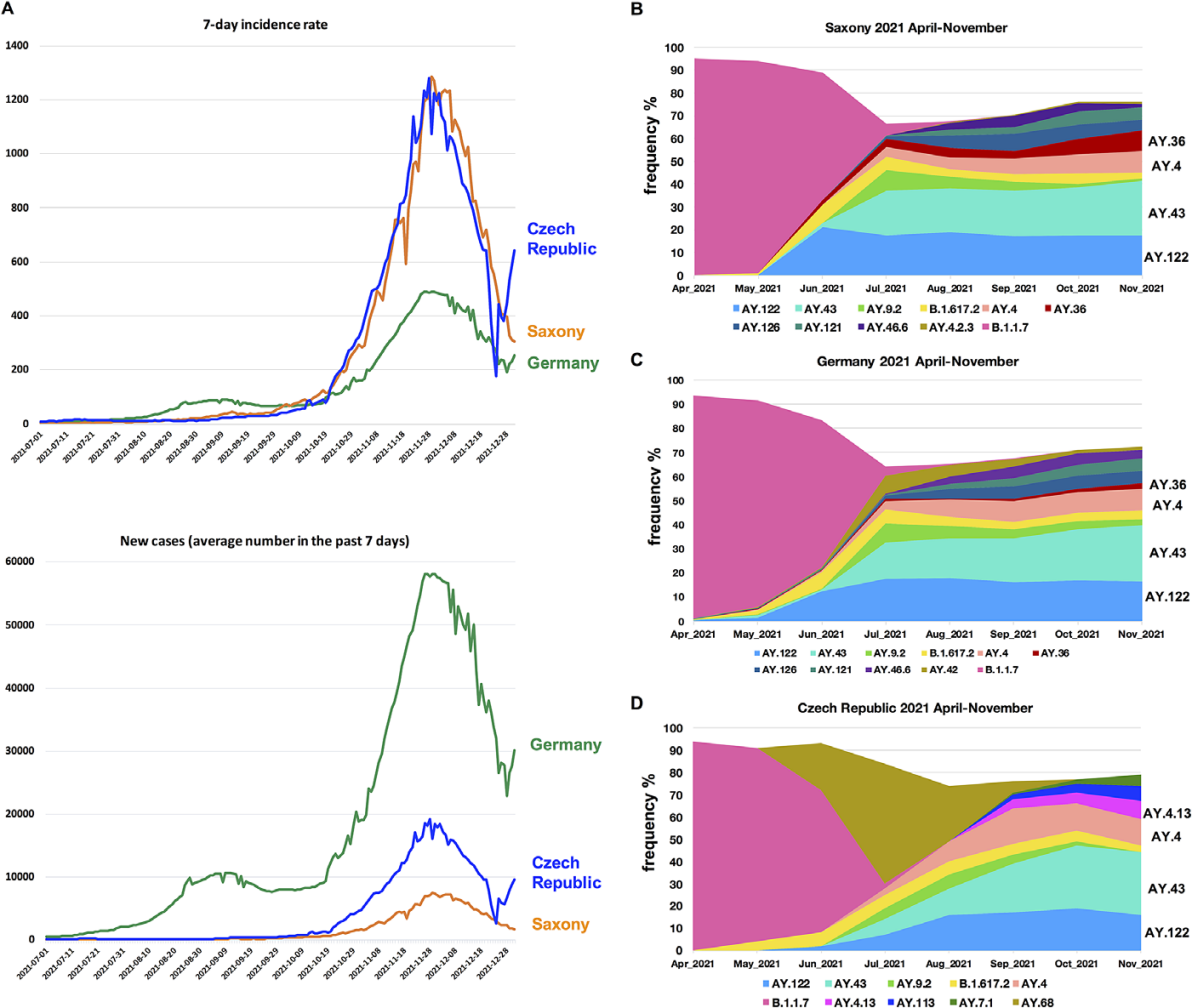
The COVID-19 pandemic waves in late 2021 in Saxony, Germany and the Czech Republic. (a) COVID-19 7-day incidence rate per 100 000 inhabitants and number of new cases (average number in the past 7 days in the total local population) are shown for each region between July and December 2021. (b–d) Lineage dynamic changes in Saxony, Germany and Czech Republic, April–November 2021. The frequency of detection (%) of each SARS-CoV-2 lineage in each month in (b) Saxony, (c) Germany, and (d) Czech Republic is displayed. To achieve a better resolution, lineages with a highest frequency < 5% during this period in each place are in most cases not separately shown, while collectively shown as the white blank space in the area plots.

In summer and early autumn 2021, the 7-day incidence rates (the normalized value of the new case number in the past 7 days based on local population) of COVID-19 in Saxony were below 10 during most time, which was much lower compared to the average incidence rates in Germany at that time (Figure 1a). However, from October on, new COVID-19 cases and the incidence rate in Saxony increased dramatically (Figure 1a). At the beginning of November, the incidence rate already exceeded 500, which was two times higher than the average value in Germany, and the severe burdens on the healthcare system resulted in a partial lockdown. It is therefore important to analyze what happened in Saxony in late autumn, that is, in September and October, that led to the high incidence in winter.

The global objective of this study is to understand how a pandemic wave got initiated and further developed (here it refers to the 2021 winter wave in Saxony), which is important for future pandemic prevention and preparation. This objective can be divided into specific objectives of addressing the following five questions:

1. What kind of SARS-CoV-2 variants were responsible for most infection cases during the 2021 winter wave?
2. Besides directly imported infection cases through travelling, did community transmission play an important role in incidence increase?
3. Under the condition of without non-pharmaceutical intervention, were there any common trends for community transmission caused by imported variants?
4. If there were locally occurring variants, were there differences in transmission pattern between locally occurring variants and imported variants?
5. Was the development course of the winter wave related with functional features of specific virus variants?

## Methods

We have sorted the methods part into three big sections. The first section includes methods performed with the dataset combining the sequences of local samples produced by us (collected from Saxony and the neighbour regions in the Czech Republic and Poland) and GISAID downloaded sequences produced by other teams; the second section includes analyses performed only with local samples produced by us; and the last section includes methods for virus functional analyses.

### Monitoring virus evolution and transmission through genomic epidemiology investigation in a global background

#### Establishment of genome sequence data set for genomic epidemiology investigation and monitoring emerging variants in a global background

We combined SARS-CoV-2 sequences generated from samples collected in a border region between Germany, Poland and the Czech Republic, with full-length SARS-CoV-2 sequences periodically downloaded from GISAID [5] to build up genome sequence data set for epidemiology investigation and monitoring emerging variants (locally generated sequences were shared on GISAID as well). We performed quality check and filtered out low-quality sequences that met any of the following criteria: (1) sequences with less than 90% genome coverage; (2) genomes with too many private mutations (defined as having >24 mutations relative to the closest sequence in the reference tree); (3) genomes with more than 10 ambiguous bases; and (4) genomes with mutation clusters, defined as 6 or more private differences within a 100-nucleotide window. These are the standard quality assessment parameters utilized in NextClade (https://clades.nextstrain.org) [6]. In the current study, between April 2021 and November 2021, around 230 200 sequences from Germany (among which around 17 500 sequences from Saxony) and 16 100 sequences from the Czech Republic were used in the genomic epidemiology analyses.

#### Lineage classification

We used the dynamic lineage classification method through the Phylogenetic Assignment of Named Global Outbreak Lineages (PANGOLIN) software suite (https://github.com/hCoV-2019/pangolin) [7]. This is intended for identifying the most epidemiologically important lineages of SARS-CoV-2 at the time of analysis [8].

#### Phylogeny and phylogeographical analyses of SARS-CoV-2

We carried out phylogenetic and phylogeographical analysis to monitor virus genetic changes and infer the transmission routes of AY.36.1 in Europe [9] with a custom build of the SARS-CoV-2 NextStrain build (https://github.com/nextstrain/ncov) [10]. The pipeline includes several Python scripts that manage the analysis workflow. Briefly, it allows for the filtering of genomes, the alignment of genomes in NextClade (https://clades.nextstrain.org) [6], phylogenetic tree inference in IQ-Tree [11–13], tree dating [14] and ancestral state construction and annotation. To infer the transmission routes of AY.36.1 in Europe, only samples fulfilling these criteria on GISAID were included in the analysis: 1. With complete sample collection dates; 2. With a complete sequence (>29 000nt) and less than 5% Ns; 3. With all the definition mutations of AY.36.1 including signature mutations of AY.36 (S: 1104L, orf1b:721R, orf1b:1538L) and four other AA substitutions: orf9b: 3S, orf3a: 223I, orf1a: 944L, N: 6L. The phylogeny analysis is rooted by *Wuhan*-Hu-1/2019 (GISAID Accession ID: EPI_ISL_402125).

#### Epidemiology data

We analyzed daily cases of SARS-CoV-2 in the Czech Republic from publicly released data provided by the Ministry of Health of the Czech Republic (https://onemocneni-aktualne.mzcr.cz/covid-19), and 7-day incidence rates per 100 000 inhabitants were calculated accordingly based on the local population. The data of 7-day-incidence rates per 100 000 inhabitants in Germany or in Saxony were obtained from the Robert Koch Institute (https://www.rki.de/DE/Content/InfAZ/N/Neuartiges_Coronavirus/Fallzahlen.html).

### Genomic epidemiology analyses of self-collected samples

#### Establishment of genome sequence data set for the detection of local transmission clusters

To detect local transmission clusters, we also performed genomic epidemiology analysis only with self-collected samples with documented metadata. In the German side, usually 5–10% of local SARS-CoV-2 positive samples were sequenced, ranging from 50 to 300 samples per week; In the Czech Republic, usually at least 50 samples per week were sequenced. The geographic coverage of sample collection in this study is shown in Supplementary Figure S1. An unbiased sampling procedure was applied to sequencing sample collection without pre-selection of the samples. Sequence quality check and lineage classification are the same as described in the previous section. Between April 2021 and November 2021, around 2 500 sequences from Germany (mainly from Saxony), and around 1 300 sequences from the Czech Republic (mainly from Ustecky Kraj) were used in the current study.

#### Identification of transmission clusters

For transmission cluster identification, phylogeny and phylogeographical analyses were performed in a similar setting as described in the previous section, but the phylogenetic tree was rooted with ‘least-squares’ methods [15] to make the phylogeny less affected by square errors of the branch lengths and therefore more accurately reflect sample-to-sample relationship. Part of the locally sequenced samples have voluntarily provided contact information. Based on experience data of samples with contact information, maximal two AA mutations between two samples from people with trackable contact were detected, and all samples of identified transmission clusters fit into this criterion with most neighbour samples only differing by zero to one AA mutation, indicating closely related transmission relationships. Also, the identified clusters were consistent with all the available contact information.

#### Phylodynamic analysis of transmission chains

Phylodynamic approaches to track the dynamic changes of transmission clusters were carried out by integrating phylogenetic and demographic information, primarily using RStudio v1.3.1093 with multiple R software [16, 17], for example, tidyverse, ggplot, and ggmap.

### Functional evaluation of local variants of interest

#### Relative growth advantage

We analyzed SARS-CoV-2 sequences from Germany that were uploaded to GISAID with complete sample collection dates from 1 October to 30 November 2021. A logistic regression model was used to estimate the relative growth advantage of certain variant compared to co-circulating variants as previously reported [18–21]. The model assumes that the increase or decrease of the proportion of a variant follows a logistic function, which is fit to the data by optimizing the maximum likelihood to obtain the logistic growth rate in units per day. Based on that, an estimate of the growth advantage per generation is obtained (assuming the growth advantage arising from a combination of intrinsic transmission advantage, immune evasion, and a prolonged infectious period [22], and the relative growth advantage per week (in percentage; 0% means equal growth) is reported. The relative growth advantage estimate reflects the advantage compared to co-circulating variants in the selected country and time frame.

#### Viruses

All viruses used were patient isolates cultured from nasopharyngeal swabs. Virus stocks were grown on Vero E6 cells in DMEM GlutaMAX supplemented with 10% FBS, 1% non-essential amino acids and 1% penicillin/streptomycin. The second passage of each virus isolate was used for experiments. The virus isolates BA.1 (hCoV-19/Germany/SN-RKI-I-405124/2021, EPI_ISL_8237557), B.1.1.7 (hCoV-19/Germany/SN-RKI-I-178035/2021, EPI_ISL_2634728), B.1.177 (hCoV-19/Germany/SN-RKI-I-017381/2021, EPI_ISL_1147543), AY.122 (hCoV-19/Germany/SN-RKI-I-348308/2021, EPI_ISL_7101815), AY.36.1 (hCoV-19/Germany/SN-RKI-I-290321/2021, EPI_ISL_5402822), were used in the virus neutralization assay and growth kinetics measurement.

#### Virus neutralization assay

All sera were derived from healthy individuals following vaccination with triple doses of BNT162b2 (around 1 month after the third shot). A 2-fold dilution series of each serum was prepared in PBS+ (supplemented with 0.3% bovine albumin, 1 mM MgCl_2_ and 1 mM CaCl_2_) and each serum concentration was incubated with 50 PFU of AY.36.1, AY.122, BA.1, B.1.1.7 or B.1.177 for 1 h at 37 °C. Confluent Vero E6 cells seeded the day before were infected with the virus-containing serum dilutions for 1 h at 37 °C and 5% CO_2_ with occasional shaking. The inoculum was aspirated, cells washed with PBS and subsequently overlayed with semi-viscous Avicel Overlay Medium (double-strength DMEM, Avicel RC-581 in H2O 0.75%, 10% FCS, 0.01% DEAE-Dextran and 0.05% NaHCO_3_). After 3 days, cells were stained with 0.1% crystal violet in 10% formaldehyde and plaques were counted. Eleven dilutions of each serum were tested. The neutralization assay was performed in three independent experiments with each serum. Each experiment was conducted in technical duplicates of each serum. Technical duplicates were averaged before further calculations. ID_50_ values were calculated using fourth-order nonlinear regression curve fits with GraphPad Prism 9.

#### Virus growth kinetics

Calu 3 cells were seeded 3 days prior to infection. On the day of infection, cells were infected with AY.122, AY.36.1 or BA.1 at MOI 0.1 diluted in PBS+ (PBS supplemented with 0.3% BSA and 1 mM MgCl_2_ and 1 mM CaCl_2_) for 1 h at 37 °C and 5% CO_2_ with occasional shaking. Afterwards, the inoculum was aspirated, the cells were washed with PBS and fresh medium (DMEM GlutaMAX supplemented with 10% FBS, 1% non-essential amino acids, 1% sodium pyruvate and 1% penicillin/streptomycin) was added. Supernatants were removed at 8, 16, 24, 48 h post infection (hpi). Infectious virus particles in the supernatant were determined using plaque assay, which was performed analogously to the neutralization assay from the infection step onwards. Results are given as plaque-forming units (PFU) per ml. Graphs were generated using GraphPad Prism 9.

### Ethical statement

The ethics committee/IRB of TU Dresden waived ethical approval for this work mainly due to the urgent need to understand SARS-CoV-2 spreading under the pandemic conditions, and no subject follow-up was conducted.

## Results

### The spreading of a few specific delta variants accompanied the high incidence rates in a cross-border region between eastern Germany and the Czech Republic

We first investigated the predominant SARS-CoV-2 variants responsible for the high COVID-19 incidence in Saxony in the autumn and winter of 2021. Between October to November 2021, four SARS-CoV-2 Delta sub-lineages were most frequently detected in Saxony: AY.122, AY.43, AY.4, and AY.36, indicating these variants caused most infections during that period (Figure 1b). The lineage dynamic changes in Germany overall were pretty similar to that in Saxony, with the exception of AY.36, which was detected in a much lower frequency compared to it in Saxony (Figure 1c). In the neighbour country, Czech Republic, the predominant variants were slightly different, with AY.122, AY.43, AY.4, and AY.4.13 accounting for most infections (Figure 1d). The analyses for the three places were based on information from GISAID acquired on 31 January 2022.

Among these lineages, AY.122 had been reported to be the dominant Delta sub-lineage in Russia and a few other Eastern European countries since April 2021 [23]. The AY.43 lineage had been detected in multiple European countries since early 2021. AY.4 was distributed worldwide, and also frequently detected in Europe, especially in UK, since April 2021 [24]. In Saxony, these several lineages were not frequently detected until late June 2021 (Figure 1b). This suggested that most cases related with these several lineages (AY.122, AY.43, and AY.4) were caused by either directly imported variants or community transmission clusters derived from the imported variants (if the further transmission of the imported cases had not been prevented).

### Several community transmission clusters of the Delta variant could be identified through genomic epidemiology

**Figure 2.**
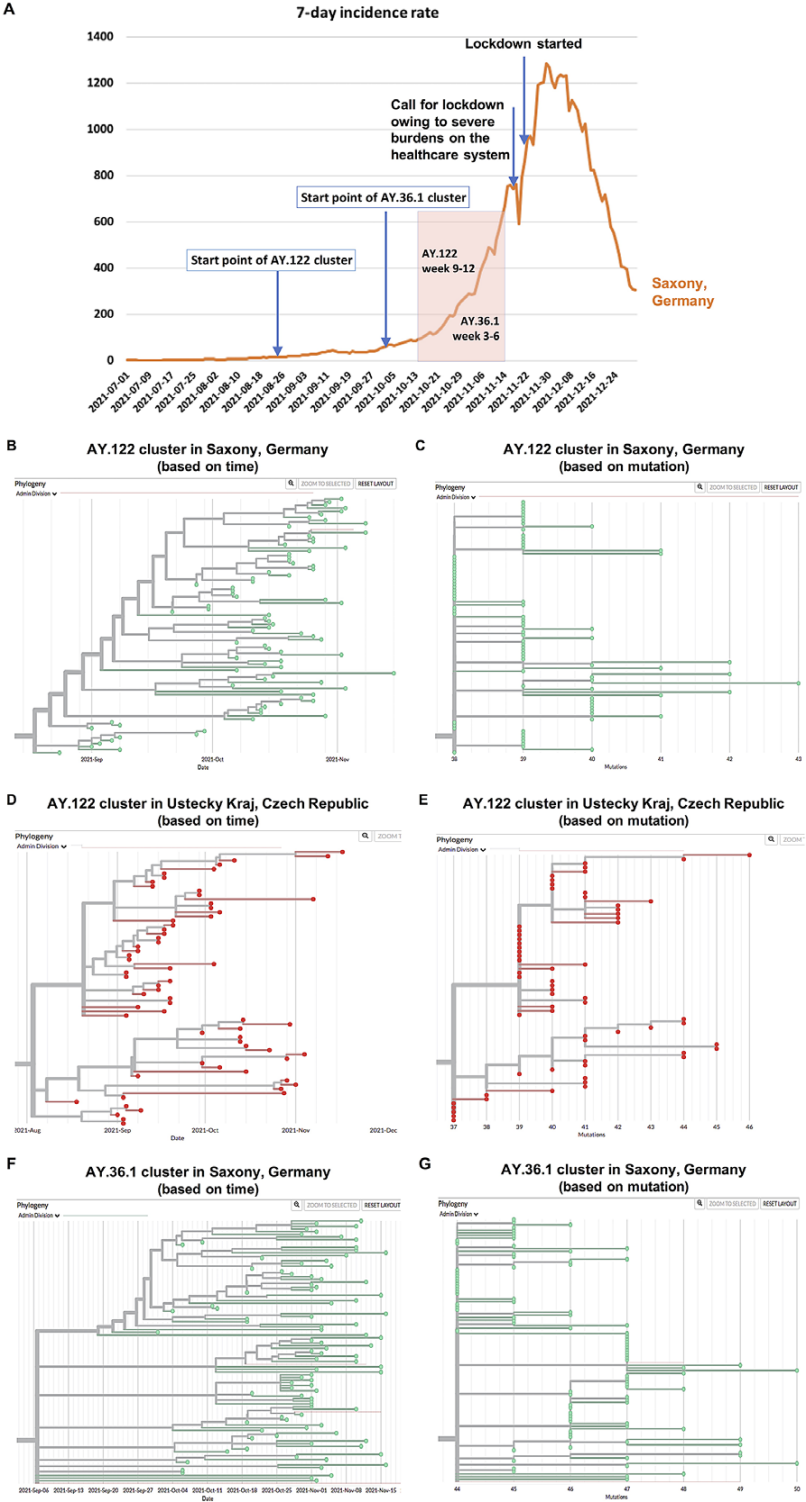
Multiple community transmission clusters were identified through genomic epidemiology, the development course of which was associated with the development course of the winter wave. (a) The association of the course of the community transmission clusters with the development course of the winter wave in 2021 in Saxony, Germany. (b,c) AY.122 cluster in Saxony, Germany is displayed based on time (b) and mutation (c), respectively. (d,e) AY.122 cluster in Ustecky Kraj, Czech Republic is displayed based on time (d) and mutation (e), respectively. (f,g) AY.36.1 cluster in Saxony is displayed based on time (f) and mutation (g), respectively. These clusters are displayed with samples collected till middle of November 2021 before the partial lockdown with each dot representing one sample.

With phylogenetic and phylogeographic analyses, genomic epidemiology may serve as a powerful tool for identifying transmission relationships under the condition of lack of contact information [3]. In this study, through genomic epidemiology analyses, we were able to identify several community transmission clusters in Saxony since August 2021 (before August no detectable prolonged transmission clusters of Delta variants), including clusters caused by AY.122, AY.43, AY.36 (later named as AY.36.1, with details described in the later section) and a few others, most of which started during or shortly after the travel season (mainly the summer holiday season) (Supplementary Figure S2). Because self-collected samples contained corresponding metadata, we performed further analyses to inspect the details of identified transmission clusters with samples collected till middle of November before the partial lockdown (Figure 2a). As a representative cluster, the AY.122 cluster kept on from August 2021 through autumn and winter 2021 (Figure 2b,c). From the neighbour region Ustecky Kraj in the Czech Republic, one AY.122 community transmission cluster was also detected, which similarly started in August 2021 (Figure 2d,e). In Saxony, a big cluster also formed through the transmission of the AY.36 variant since October 2021 (Figure 2f,g).

### Dynamic changes of the two AY.122 clusters display a highly similar pattern

**Figure 3.**
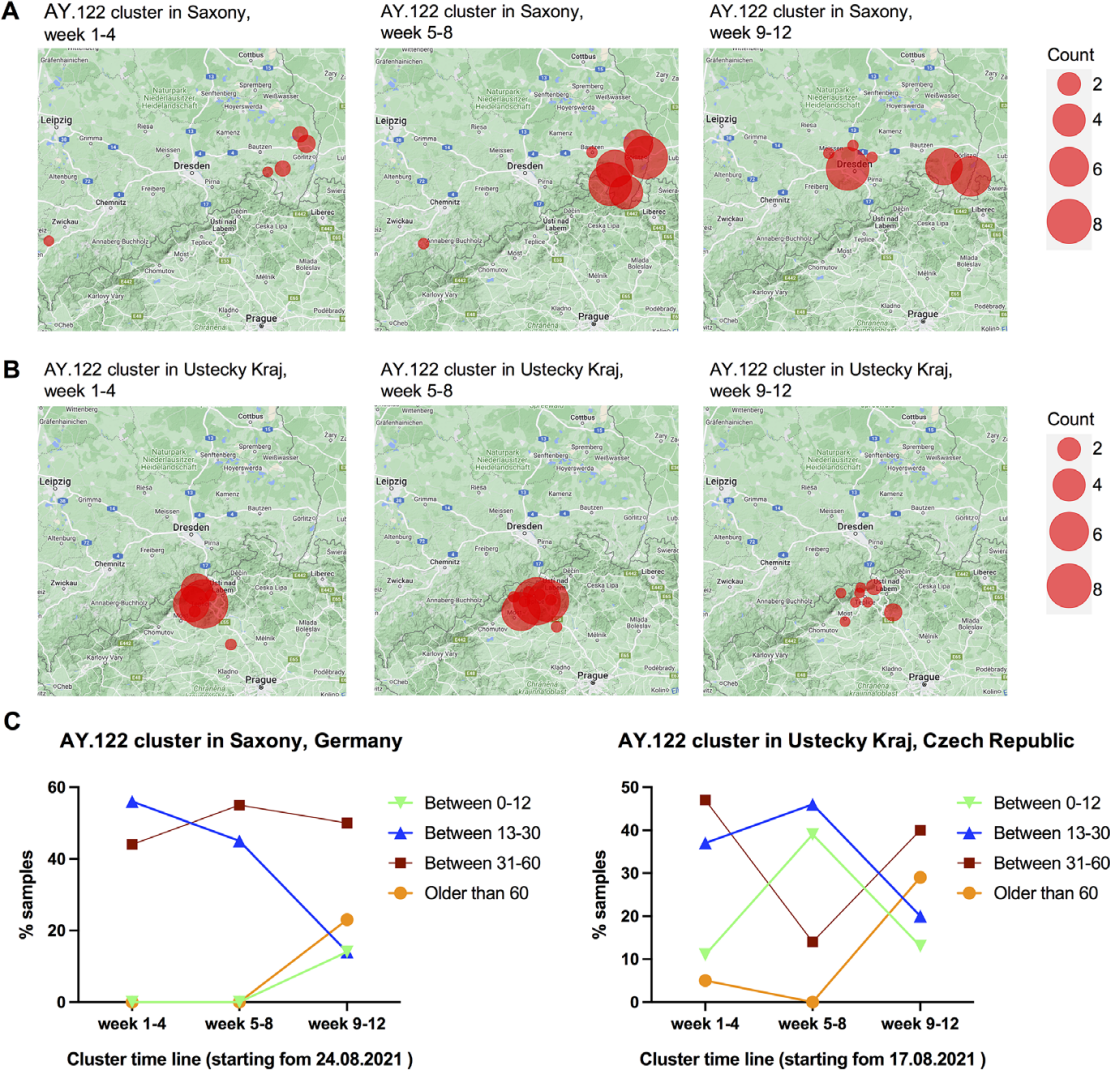
Dynamic changes of geographic distribution and age distribution of detected new samples in each cluster in every 4 weeks along with the cluster timeline. (a,b) The geographic distribution of detected new samples in the AY.122 cluster in Saxony, Germany (a) and the AY.122 cluster in Ustecky Kraj, Czech Republic (b) every 4 weeks along with the cluster timeline is displayed based on documented postcodes. (c) The age distribution of detected new samples in each cluster in every 4 weeks is displayed. In both clusters, the ratio of patients aged above 60 years highly increased after 8-week community transmission.

With available metadata, we performed more detailed analysis of the AY.122 clusters in both Saxony, Germany, and Ustecky Kraj, Czech Republic. The samples in one community transmission cluster are often geographically clustered at the early stages of the cluster development [24, 25]. Consistent with that, we observed clear geographic clustering in both two AY.122 clusters. As shown in Figure 3a,b, both AY.122 clusters in the two places displayed a similar transmission pattern in the dynamic changes of geographic distribution. In weeks 1–4, the virus mainly spread locally in a small region; in weeks 5–8, more people were infected, but still mainly in a small local region; in weeks 9–12, the virus got more widely spread to the surrounding regions, or even reached further to more remote places such as for the AY.122 cluster in Saxony.

With metadata of patient age, we also analyzed the dynamic changes of age distribution in these two AY.122 clusters. These two clusters also displayed a highly similar pattern in the dynamic change of age destitution (Figure 3c). In weeks 1–4, mainly young to middle-aged people (<60 years old) got infected; in weeks 5–8, a similar age distribution as weeks 1–4 was detected; in weeks 9–12, the ratio of people aged above 60 years clearly increased and went up to about 30% of the newly infected population, which was much higher than that in the first 8 weeks (0–5%). This indicated that in both clusters, the ratio of elder patients highly increased after around 2-month community transmissions, which means a higher risk for increased healthcare burdens.

The period of weeks 9–12 of the AY.122 cluster was correlated to the period of the exponential incidence increases between the middle of October to the middle of November 2021 in Saxony (as shown in Figure 2a). This observation suggests that the limited spreading of the virus mainly among young to middle-aged people in the first 8 weeks paved a base for the wide spreading of the virus later.

### Community transmissions caused by a locally occurring variant show a distinct transmission pattern

**Figure 4.**
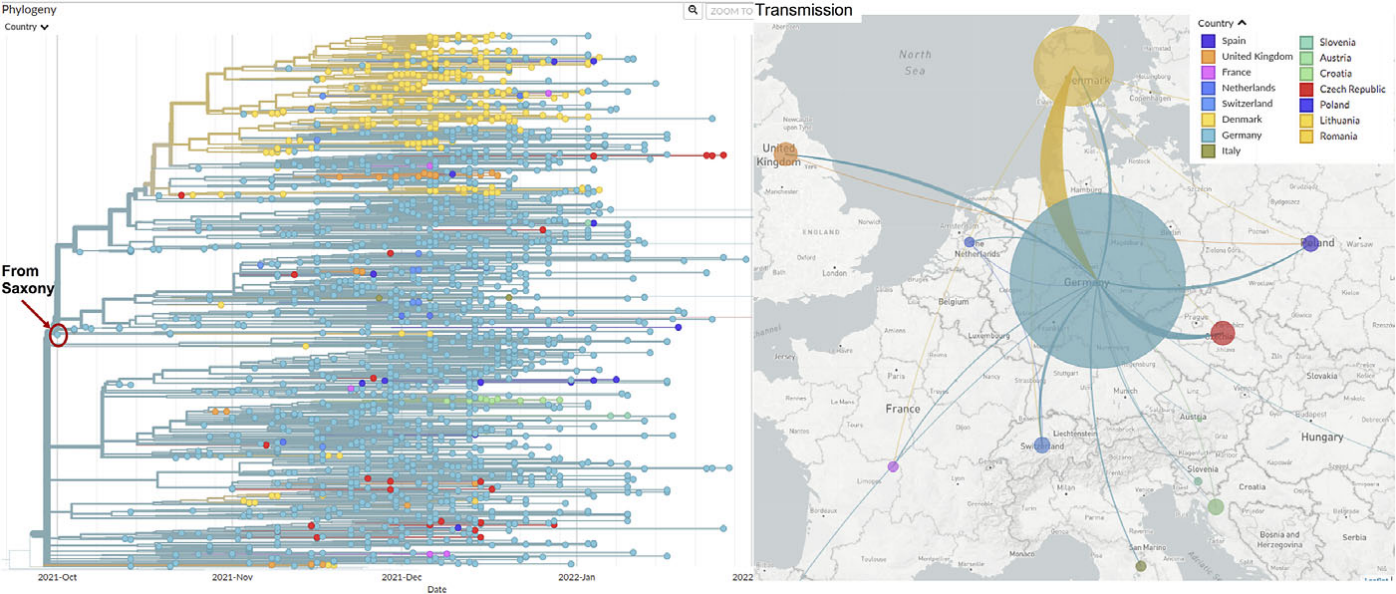
Transmission routes of AY.36.1 in Europe are inferred based on phylogeny analysis. The size of the circle represents the number of genomes from all AY.36.1 in each country collected by the end of January 2022. The line colours correspond to the exporting locations. Left: Phylogeny tree of AY.36.1, with branch length representing time. The first sample was detected in Saxony, Germany in early October 2021; right: estimated transmission routes of AY.36.1 in Europe.

In Saxony, one of the predominant variants responsible for many infection cases was AY.36. Through detailed phylogenetic analyses we discovered that most local AY.36 samples belong to a specific, locally occurring variant derived from virus evolution of AY.36. This special AY.36 grew rapidly in Saxony since October and formed a big cluster. Compared with originally defined AY.36 (with signature mutations S: 1104L, orf1b:721R, orf1b:1538L), most local samples carried four additional AA substitutions: orf9b: 3S, orf3a: 223I, orf1a: 944L, N: 6L. We reached an agreement with other genomic epidemiologists and defined these particular AY.36 samples as a new PANGOLIN lineage of Delta variant AY.36.1 (more information about defining this new lineage: https://github.com/cov-lineages/pango-designation/issues/434). The machine learning process of the PANGOLIN designation included some samples that do not contain all the definition mutations of AY.36.1. In this study, we only focus on AY.36.1 samples that strictly match the definition of AY.36.1 containing both the three AY.36 signature mutations and all the four extra mutations as described above. In the international background, the first AY.36.1 sample was detected in Saxony at the beginning of October, and the earliest 30 samples were almost exclusively from Saxony only with a few exceptions (Figure 4). By the end of November, it was already detected in 10 European countries and in several other continents. In December it further spread to multiple other European countries and also to other continents. Regarding transmission route, Germany and Denmark (mainly from December on) were the major source locations of AY.36.1(Figure 4).

In Saxony, the AY.36.1 variant expanded rapidly and formed a big cluster as shown in Figure 2f,g. We therefore analyzed whether this locally occurring variant shared the same community transmission pattern compared to other Delta variants.

**Figure 5.**
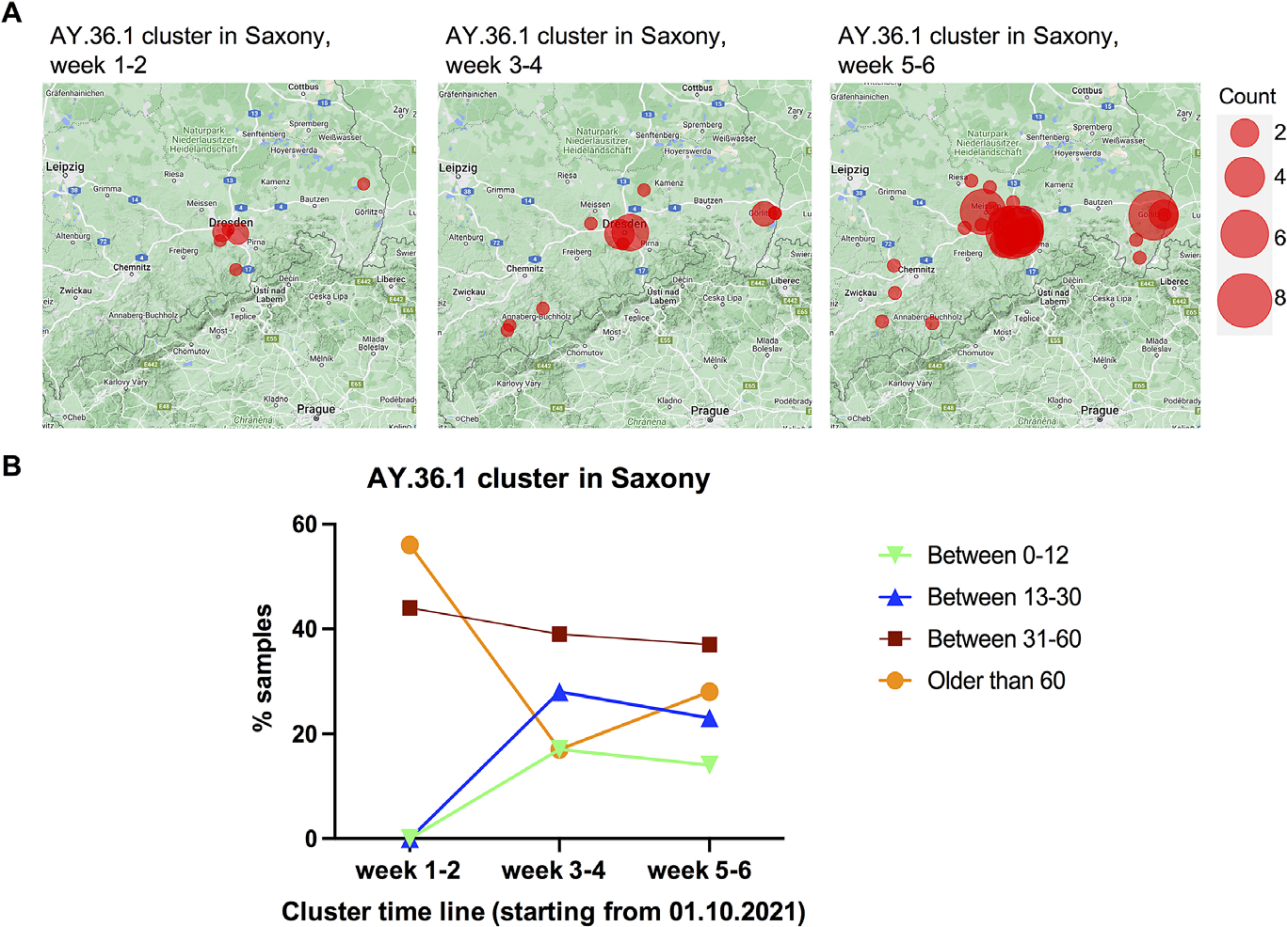
Dynamic changes of geographic distribution and age distribution of detected new samples in the AY.36.1 cluster in every 2 weeks along with the cluster timeline. (a) The geographic distribution of detected new samples in the AY.36.1 cluster in Saxony, Germany in every 2 weeks along with the cluster timeline is displayed based on documented postcodes. (b) The age distribution of detected new samples in the AY.36.1 cluster in every 2 weeks is displayed.

As the AY.36.1 cluster expanded much quicker than the AY.122 cluster, we analyzed the dynamic changes of the transmission pattern of this cluster in every 2 weeks (instead of every 4 weeks like that for the AY.122 cluster) starting from the first sample identified at the beginning of October. The period of weeks 3–6 of the AY.36.1 cluster was correlated to the period of the exponential incidence increases between the middle of October to the middle of November 2021 in Saxony (Figure 2a). In the first 2 weeks, similar to the AY.122 clusters, the AY.36.1 cluster was also mainly spreading in a relatively small local region showing the feature of geographic clustering (Figure 5a). But from the third week on, it quickly expanded to the surrounding regions in one explosive way. By the end of the sixth week, it was already detected in lots of regions in Saxony. Regarding age distribution changes, there were many people aged above 60 years being infected by this variant in weeks 1–2 already, indicating a risk of escalating healthcare burdens (Figure 5b). These results revealed that the transmission pattern of this cluster caused by the local variant AY.36.1 was different from the transmission pattern of the two AY.122 clusters caused by imported variants.

### Comparative analyses of virus propagation and antibody neutralization between AY.36.1 and other VOCs

**Figure 6.**
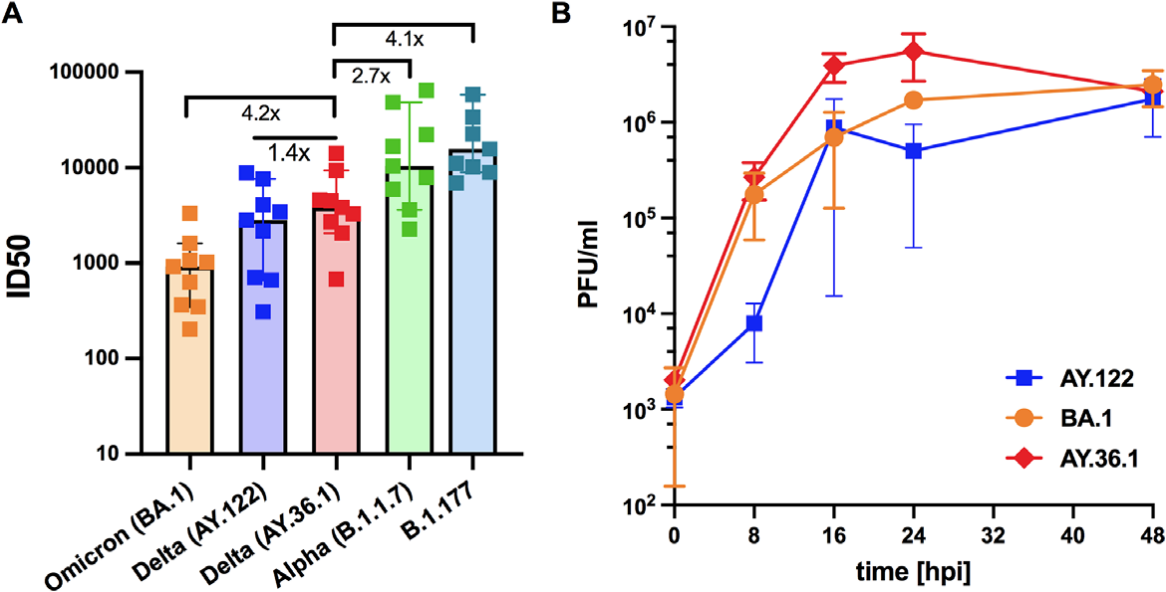
Neutralization efficacy and growth kinetics of AY.36.1 in comparison with multiple other SARS-CoV-2 variants. (a) Neutralization efficacy of sera from individuals following vaccination with triple doses of BNT162b2 (*n* = 9, BNT162b2) against active virus of several VOCs variants including the Alpha (B.1.1.7), Delta (AY.122 and AY.36.1), and Omicron (BA.1) variants. B.1.177 is a non-VOC variant wildly spread in winter 2020 and early 2021. ID_50_, the serum dilution required for 50% virus inhibition. Bars represent the median ID_50_ values with 95% confidence interval. (b) Growth kinetics comparing AY.36.1 with AY.122 and Omicron variant BA.1 on Calu 3 cells as titrated by plaque assay. All data represent at least two independent experiments, each with two technical replicates.

The estimated growth rate of AY.36.1 in Germany between October and November 2021 was above most other co-circulating Delta variants, as manifested by a 32% relative growth advantage of the AY.36.1 compared to co-circulating variants (0% means equal growth) (Supplementary Figure S3). To verify if the advantage in growth rate was related to a functional feature of this variant, we evaluated the antibody neutralization and virus propagation abilities of this variant in comparison with a few other variants circulating in Germany in 2021. We compared AY.36.1 with another Delta sub-lineage AY.122, the Alpha lineage B.1.1.7, the Omicron sub-lineage BA.1, and one non-VOCs B.1.177, which was one of the predominant lineages during the second wave in winter 2020 and early 2021 [26], by testing their susceptibilities to vaccine-elicited serum neutralizing antibodies in individuals following vaccination with triple doses of BNT162b2. Consistent with other reports [27–35], these experiments showed a decrease of neutralization sensitivity of the Delta sub-lineages (both AY.36.1 and AY.122) compared to the Alpha variant (B.1.1.7) and the non-VOCs B.1.177, but higher sensitivity compared to the Omicron variant (BA.1) (Figure 6a). The two Delta sub-lineages AY.122 and AY.36.1 displayed similar neutralization sensitivity (Figure 6a).

As the circulation period of AY.122 and BA.1 in Saxony overlapped with AY.36.1, we compared the replication ability of AY.36.1 with AY.122 and BA.1. For that, we infected lung epithelial cell line Calu-3 with these three variant isolates. AY.36.1 showed a clear replication advantage compared to the Delta sub-lineage AY.122, especially in the first 24 h after infection, but no clear advantage compared to the later dominating Omicron sub-lineage BA.1 (Figure 6b). These data support higher replication rate of AY.36.1, corresponding to faster spread of it over other co-circulating Delta sub-lineages during the same period.

## Discussion

This study has revealed that long-lasting community transmission clusters developing since early autumn contributed to the incidence surge in late autumn and winter 2021. These clusters were mostly formed by community transmission of variants imported during the summer and early autumn vacation time. In addition to imported variants, one locally occurring new variant AY.36.1 with replication advantages played an important role as well in driving the local pandemic development possibly with an acute effect.

In this study, with available metadata, we were able to investigate the transmission pattern of community transmission clusters. Analysis of two representative long-lasting AY.122 community transmission clusters in Saxony, Germany and Ustecky Kraj, Czech Republic revealed a similar transmission pattern in the dynamic changes of geographic distribution and age distribution. In particular, a shift in age distribution that the ratio of people aged above 60 years increased in the later stages of community transmission was observed, which is consistent with epidemiology data that more cases of COVID-19 in elder people were often observed in the later stages of an epidemic [36], and also corresponding to the results of another investigation in USA that the 2020 autumn-winter wave in the United States was mainly driven by adults 20 to 49 years of age, with this age group contributing substantially to virus transmissions for the development of that autumn-winter wave [37].

The cluster derived from the locally occurring variant AY.36.1 displayed a distinct transmission pattern: geographically much more widely spread in a short time and more elder people got infected from the very beginning. This means the consequence of the spreading of this variant could be: many elder people got infected in a short time, which may impose big pressure on the healthcare system, and this was what actually happened in Saxony in late 2021.

In line with multiple previous reports (e.g., [27–35]), functional analyses revealed that the Delta sub-lineages (AY.122 and AY.36.1) were more resistant against antibody-mediated neutralization compared to the Alpha variant (B.1.1.7), but much more sensitive in comparison with the Omicron variant (BA.1). The two Delta sub-lineages AY.122 and AY.36.1 had similar neutralization sensitivity, but AY.36.1 showed a clear replication advantage compared to AY.122. Our findings indicate that immune evasion of AY.36.1 is similar to other Delta sub-lineages, suggesting that increased human-to-human transmissibility (e.g., due to increased replication in the upper respiratory tract or augmented infection of cells) might contribute to the expansion of AY.36.1. All the Delta sub-lineages were outcompeted by the Omicron variant at the beginning of 2022 [38]. As also reported by several other studies [28, 29, 31, 35, 39, 40], the robust neutralization evasion by the Omicron variant indicates that the Omicron variant is more adept than the Delta to spread in populations that are vaccinated, which explains the takeover of AY.36.1 by the Omicron variant despite the early Omicron variant BA.1 has no clear replication advantage compared to AY.36.1.

Our investigation indicates that locally occurring new variants with a replication advantage or reduced sensitivity to antibody might be able to cause a significant impact on the local pandemic development, which emphasizes the importance of regular genomic epidemiology analysis and mutation surveillance. To make the surveillance more real-time, it will be beneficial to reduce the turnaround time between sampling and sequence acquirement.

In this investigation, we were basically looking into this pandemic wave through one microscope. Information acquired through this method concurs with information achieved through analysis of large-scale data, but with more details [36, 37, 41]. This is also a quite rare opportunity that we could perform the same analysis with data collected from two countries. Therefore, the information is least affected by local population, policy, difference in medical system and other factors, and might reflect a more general pattern for community transmission clusters derived from imported variants without non-pharmaceutical intervention.

Overall, our investigation indicates, to prevent severe burdens on healthcare systems caused by a sharp increase of COVID-19 incidence or the performance of certain mutant variants, it is important to detect possible emerging new variants at the earliest possible time through regular genomic epidemiology analyses. These data suggest that mitigation approaches should be more effective if taken in the earlier stages of community transmission, such as by offering free tests to break the transmission chains. This could prevent or at least mitigate a dramatic incidence increase later. Furthermore, information provided by virus genomic epidemiology and genomic surveillance, as well as other surveillance measures such as wastewater surveillance, may inform public health strategy and facilitate the accomplishment of appropriate prevention/mitigation actions and therefore help keep normal daily life in most time.

### Limitations of this study

In this study, we focus on SARS-CoV-2 virus transmission in a cross-border region between Germany, Poland and the Czech Republic. Therefore, this is a relatively small-scale study.

### Strengths of this study

Multiple virus genomic epidemiology approaches and virus functional analyses have been applied in this study, so the winter wave development can be evaluated in a relatively comprehensive way. Also, this is a quite rare opportunity that we could perform the same analysis with data collected from two countries. Therefore, the information might reflect a more general pattern for community transmission clusters derived from imported variants without non-pharmaceutical intervention.

## Supporting information

Yi et al. supplementary material Yi et al. supplementary material

## Data Availability

A list of GISAID accession ID for AY.36.1 samples that strictly match the definition of AY.36.1 (as described at https://github.com/cov-lineages/pango-designation/issues/434 containing both the three AY.36 signature mutations (S: 1104L, orf1b:721R, orf1b:1538L) and the four extra mutations of AY.36.1 (orf9b: 3S, orf3a: 223I, orf1a: 944L, N: 6L)) is provided at https://github.com/genomesurveillance/delta-variant-sublineage. The processed SARS-CoV-2 AY.36.1 genome data in the form of phylogenetic tree are also available at https://github.com/genomesurveillance/delta-variant-sublineage. More general information about AY.36.1 genome number in each country during certain time period can be acquired by choosing the relevant location and collection period on the GISAID database (with searching items: VOC Delta (variants); Substitutions: N_P6L, NS3_T223I, NSP3_S126L, NSP3__P1469S; Complete; Low coverage excluded; Collection date complete). To access sequence data from GISAID, registration with https://www.gisaid.org/ is necessary, which involves agreeing to GISAID’s Database Access Agreement. Biological materials (i.e., virus variant isolation) generated as a part of this study will be made available but may require execution of a materials transfer agreement. Data processing and visualization were performed using publicly available software, primarily RStudio v1.3.1093. Code for constructing phylogenetic maximum likelihood (ML) and time trees as well as phylogeographic analyses is available at https://github.com/genomesurveillance/delta-variant-sublineage, which is modified from SARS-CoV-2-specific procedures github.com/nextstrain/ncov.

## Supplementary material

For supplementary material accompanying this paper visit http://doi.org/10.1017/S0950268824000761. click here to view supplementary material
