# Supplementary material for: Investigating the cause of a 2021 winter wave of COVID-19 in a border region in eastern Germany: a mixed-methods study, August to November 2021
Source: Epidemiol Infect. 2024 May 16;152:e87. doi: 10.1017/S0950268824000761 (PMC11149030; doi:10.1017/S0950268824000761)
Supplement: Yi et al. supplementary material [file S0950268824000761sup001.docx]

*Epidemiology and Infection*

**Investigating the cause of a 2021 winter wave of COVID-19 in a border region in Eastern Germany: a mixed-methods study, August to November 2021**

Buqing Yi^1*^, Eva Patrasová^2,3^, Lenka Šimůnková^2^, Fabian Rost^4,5^, Sylke Winkler^6^, Alexa Laubner^1^, Susanne Reinhardt^4^, Andreas Dahl^4^, Alexander H. Dalpke^1,7^

**Supplementary Material**


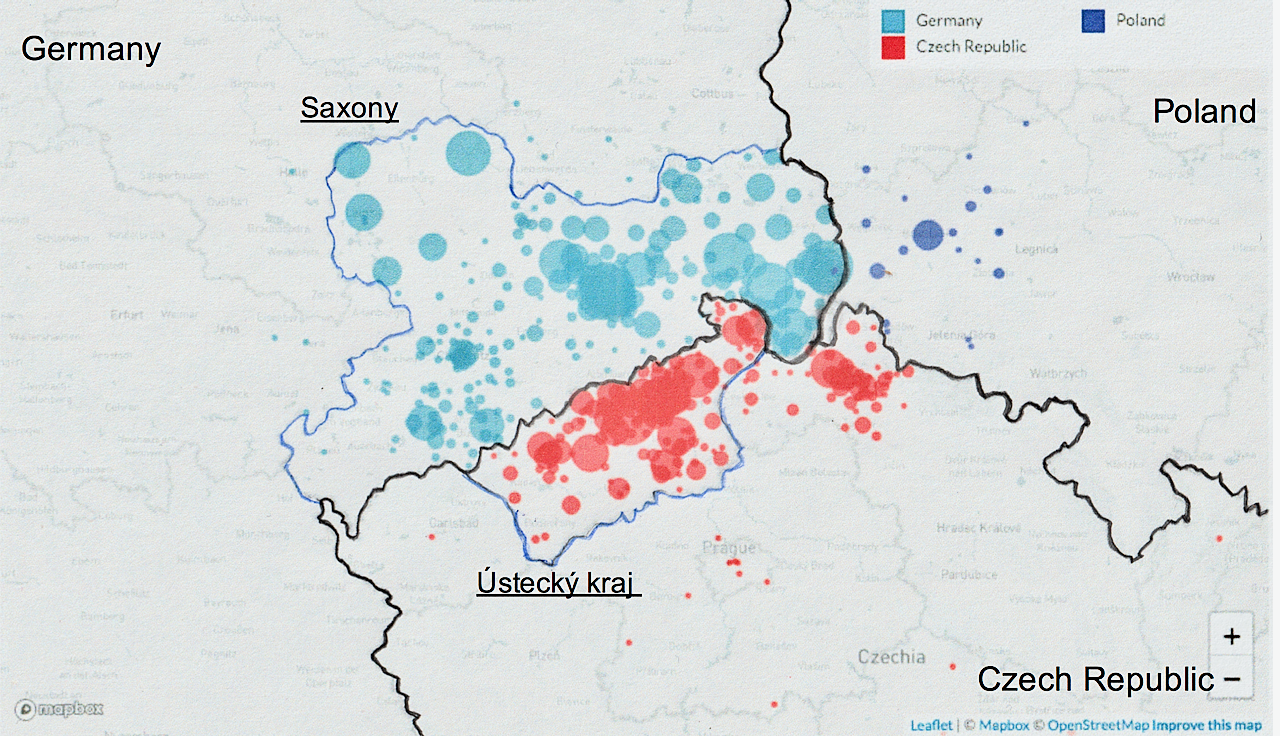


**Figure S1: Geographic coverage of self-collected samples in a border region between Germany, Czech Republic, and Poland**. Circle represents sample size of each post code.

**Figure S2: Genomic epidemiology analysis of samples from Saxony, Poland and the Czech Republic revealed multiple long-lasting community transmission clusters driving the incidence increases in late autumn and winter 2021.** Clusters detected in Saxony (Germany) and Ustecky Kraj (Czech Republic) are indicated. Polish samples were mainly collected from a drive-in test center close to the border with Saxony. Only major clusters are labelled. Most clusters started from August or September during the travel season.

**
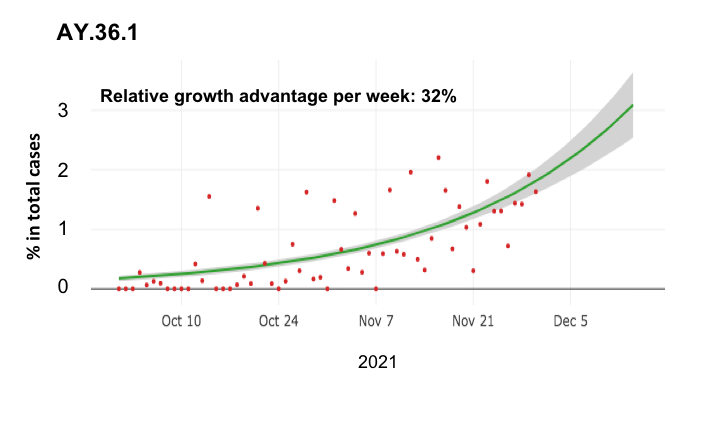
**

**Figure S3: Growth of AY.36.1 in Germany between October 1^st^ to November 30^th^ 2021.** Model fits are based on a logistic regression. Dots represent the daily proportions of variants. The relative growth advantage per week (in percentage; 0% means equal growth) is reported. The shaded areas correspond to the 95% CIs of the model estimates. The AY.36.1 spread quickly in Germany with a relative growth advantage of around 30% compared to co-circulating variants.
